# Associations Between External Radiation Doses and the Risk of Psychological Distress or Post-traumatic Stress After the Fukushima Daiichi Nuclear Power Plant Accident: the Fukushima Health Management Survey

**DOI:** 10.2188/jea.JE20210226

**Published:** 2022-12-05

**Authors:** Itaru Miura, Masanori Nagao, Hironori Nakano, Kanako Okazaki, Fumikazu Hayashi, Mayumi Harigane, Shuntaro Itagaki, Hirooki Yabe, Masaharu Maeda, Tetsuya Ohira, Tetsuo Ishikawa, Seiji Yasumura, Kenji Kamiya

**Affiliations:** 1 Department of Neuropsychiatry, Fukushima Medical University School of Medicine, Fukushima, Japan; 2 Radiation Medical Science Center for the Fukushima Health Management Survey, Fukushima Medical University, Fukushima, Japan; 3 Department of Epidemiology, Fukushima Medical University School of Medicine, Fukushima, Japan; 4 Department of Physical Therapy, Fukushima Medical University School of Health Sciences, Fukushima, Japan; 5 Department of Public Health, Fukushima Medical University School of Medicine, Fukushima, Japan; 6 Department of Disaster Psychiatry, Fukushima Medical University School of Medicine, Fukushima, Japan; 7 Department of Radiation Physics and Chemistry, Fukushima Medical University School of Medicine, Fukushima, Japan; 8 Research Institute for Radiation Biology and Medicine, Hiroshima University, Hiroshima, Japan

**Keywords:** Fukushima nuclear disaster, external radiation dose, psychological distress, post-traumatic stress

## Abstract

**Background:**

The relationship between radiation levels and mental health status after a nuclear disaster is unknown. We examined the association between individual external radiation doses and psychological distress or post-traumatic stress after the Fukushima Daiichi nuclear power plant accident in March 2011 in Japan.

**Methods:**

The Mental Health and Lifestyle Survey was conducted from January 2012. Based on the estimated external radiation doses for the first 4 months, a total of 64,184 subjects were classified into <1 mSv, 1 to <2 mSv, and ≥2 mSv groups. Odds ratios (ORs) and 95% confidence intervals (CIs) of psychological distress and post-traumatic stress, with the <1 mSv group as the reference, were calculated using logistic regression analysis adjusted for age, sex, evacuation, perception of radiation risk, and subjective health status.

**Results:**

The prevalence of psychological distress/post-traumatic stress in the <1 mSv, 1 to <2 mSv, and ≥2 mSv groups was 15.1%/22.1%, 14.0%/20.1%, and 15.0%/21.7%, respectively. In women, although the ≥2 mSv group tended to have a higher risk of psychological distress with the age-adjusted OR of 1.13 (95% CI, 0.99–1.30), the adjusted OR decreased to 1.00 (95% CI, 0.86–1.16) after controlling for all variables. On the other hand, there were no dose-dependent associations between radiation dose and post-traumatic stress.

**Conclusion:**

Although external radiation doses were not associated with psychological distress, evacuation and perception of radiation risk may increase the risk of psychological distress in women in the higher dose group.

## INTRODUCTION

The Great East Japan Earthquake of magnitude 9.0 on the Richter scale occurred on March 11, 2011, which was followed by a tsunami that led to the Fukushima Daiichi nuclear power plant accident. The accident was classified as a Level 7 emergency, the highest level on the International Nuclear Event Scale,^1^ and released radioactive elements, which forced many people living in the areas near the nuclear power plant to evacuate from their hometown and change their lifestyles.^2^ Furthermore, many people in Fukushima Prefecture experienced increased anxiety over radiation and reduced subjective well-being.

A previous report on the health effects of the Chernobyl nuclear power plant accident by the World Health Organization concluded that mental health problems are the largest public health problem caused by the accident.^3^ Post-traumatic stress disorder (PTSD) and depression, which often coexist,^4^ are the most common mental health problems after disasters.^5^ In a previous study, the prevalence of general psychological distress was high 3 years after the Fukushima Daiichi nuclear power plant accident, although the prevalence of psychological distress and post-traumatic stress decreased over time.^6^ According to a cross-sectional survey conducted in 2020, the prevalence of psychological distress, post-traumatic stress, and radiation health anxiety was high among evacuees of the Fukushima Daiichi nuclear power plant accident.^7^

A systematic review^8^ showed that various factors were associated with mental health status after natural disasters, such as disaster-related factors, coping factors, health-related factors, and personal factors. Similarly, psychological distress among evacuees after the Fukushima Daiichi nuclear power plant accident was associated with disaster-related factors (experience of the nuclear power plant accident and loss of someone close due to the disaster), personal factors (female sex, older age, and unemployment), health-related factors (history of mental illness),^9^ and perception of radiation risk.^10^^,^^11^ Moreover, the living area at the time of the disaster was also significantly related to psychological distress, and the distribution of psychological distress exhibited a pattern similar to that of the environmental radiation levels.^9^ However, the relationship between radiation levels and psychological distress or post-traumatic stress is unknown, and there are no well-designed studies regarding the association between radiation doses and psychological distress at the individual level. Therefore, we examined the association between individual external radiation doses and psychological distress or post-traumatic stress after the Fukushima Daiichi nuclear power plant accident using data from the large-scale Fukushima Health Management Survey.^12^^,^^13^

## METHODS

### Subjects and study design

**Figure 1.**
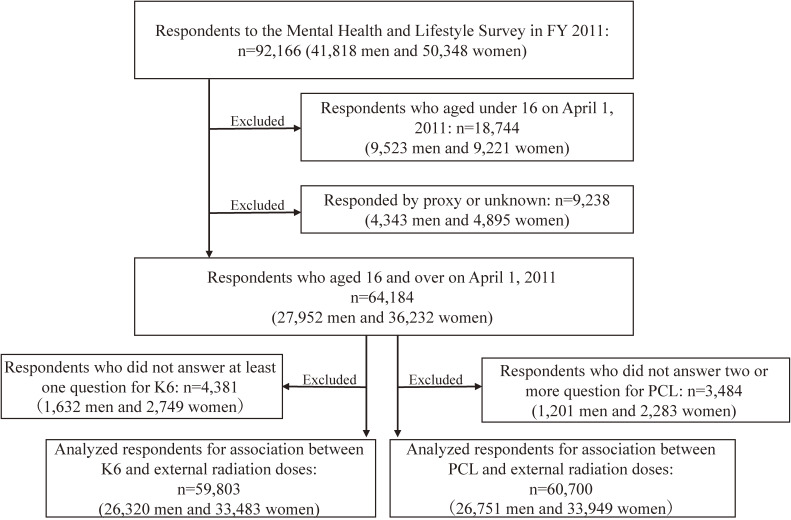
Flow chart of the study selection

The Mental Health and Lifestyle Survey, a detailed survey of the Fukushima Health Management Survey, was a questionnaire survey focusing on lifestyle and mental health and included adult residents (aged ≥16 years on April 1, 2011) of the evacuation zone designated by the Japanese government (ie, Hirono town, Naraha town, Tomioka town, Kawauchi village, Okuma town, Futaba town, Namie town, Katsurao village, Minamisoma city, Tamura city, Kawamata town, Iitate village, and part of Date city in Fukushima Prefecture),^12^^–^^14^ with a target population of 64,184 persons. Among them, we excluded 4,381 and 3,484 participants because of missing data on Kessler 6-item psychological distress (K6)^15^^,^^16^ (at least one missing item) and the PTSD Checklist Stressor-Specific Version (PCL)^17^ (at least two missing items) scores, respectively. Finally, 59,803 and 60,700 participants with available data on K6 and PCL scores were included in the analysis (Figure 1). This study was approved by the Ethics Committee of Fukushima Medical University (Nos. 1316, 1257, 1294, 1659, 2020-201, and 2020-239).

### Measures

The outcome of this study was the K6 and PCL scores. The Japanese version of the K6^16^ and PCL^18^ scale has been shown to have sufficient validity and reliability. The K6^15^^,^^16^ is composed of six questions (score range: 0–24) that assess non-specific psychological distress during the previous 30 days. We adopted a cutoff score of ≥13, which is classified as indicating probable severe mental illness.^19^ The PCL was used to assess post-traumatic symptoms, which is a 17-item self-report measure, and the cut-off was set as a PCL score of ≥44.^17^ For perception of radiation risk to genetic effects, we asked participants about their beliefs on the potential risks of radiation exposure using the following question: “What is the likelihood that the health of your future (ie, as of yet unborn) children and grandchildren will be affected as a result of your current level of radiation exposure?” on a four-point Likert scale and divided into two categories: “very unlikely” and “unlikely” or “likely” and “very likely”.^11^ Furthermore, subjective health status was obtained from five options and divided into three categories: “very good” and “good”; “passably” or “poor” and “very poor”.^11^ External radiation doses were estimated using data from the Basic Survey of the Fukushima Health Management Survey for the estimation of radiation dose, which was launched approximately 4 months after the nuclear power plant accident, and previous reports contain detailed descriptions on the methods.^20^^,^^21^ Briefly, it is a self-administered questionnaire survey that asked residents (*n* = 2,026,097) of Fukushima Prefecture to record and send back information on their behavior (including place of residence, places visited, length of time spent indoors and outdoors, and traveling time) from March 11 to July 11, 2011. The respondents’ behavior records were digitalized, and a computer program calculated individual effective doses due to external exposure by superimposing the behavior records with daily gamma ray dose rate maps drawn after the nuclear power plant accident. In total, 568,632 (27.7%) residents responded to the questionnaire by March 31, 2020. In this study, 36,401 (56.7%) subjects responded to the questionnaire, and the distributions of individual external doses of the respondents for the first 4 months were as follows: <1 mSv, 69.8%; 1 to <2 mSv, 23.1%; and ≥2 mSv, 7.0%.

### Statistical analysis

The subjects were classified into three groups according to the estimated external radiation doses—<1 mSv, 1 to <2 mSv, and ≥2 mSv. Analysis of variance, Fisher’s exact test, and the chi-squared test were used to compare the characteristics of the subjects between the groups. Since information on external radiation doses was lacking for 27,783 participants (43.3%) because of the lack of response to the Basic Survey of the Fukushima Health Management Survey, we imputed external radiation doses to make 10 datasets using multiple imputation by chained equations with predictive mean matching methods under fully conditional specification.^22^^–^^24^ In the imputation model, in addition to the estimated external radiation dose, the following variables were used as covariates: sex (no missing), age at disaster (no missing), area of residence at the time of the disaster (0.39% missing), and area of residence as half a year after the disaster (no missing). Odds ratios (ORs) and 95% confidence intervals (CIs) of psychological distress and post-traumatic stress, with the <1 mSv group as the reference, were calculated using logistic regression analysis adjusted for age at April 1, 2011 (continuous), sex, perception of radiation risk to genetic effects, subjective health status, and evacuation (defined as residents registered in the municipalities whose entire area was the evacuation zone designated by the Japanese government or self-reported experience of moved into shelters or temporary housing). The logistic regression analyses were performed for each imputed dataset, and the results were combined using Rubin’s rule.^22^ We also performed the same analyses using a non-imputed dataset for sensitivity analyses.

Data were analyzed using SAS (version 9.4; SAS Institute, Cary, NC, USA). The MI procedure was used to create multiply imputed datasets, and the MIANALYZE procedure was used to combine the results of the analyses of imputations. Significance was defined as *P* < 0.05, and all tests were two tailed.

## RESULTS

### Participant characteristics

For the total population, the prevalence of high risk of psychological distress (K6 score ≥13) and post-traumatic stress (PCL score ≥44) was 14.6% and 21.5%, respectively. The characteristics of each group according to the external radiation dose are shown in Table 1. The prevalence of K6 score ≥13 for the <1 mSv, 1 to <2 mSv, and ≥2 mSv groups was different across the groups—15.1%, 14.0%, and 15.0%, respectively (Table 1). The prevalence of PCL score ≥44 was also different across the groups—22.1%, 20.1%, and 21.7% for the <1 mSv, 1 to <2 mSv, and ≥2 mSv groups; it was lowest in the 1 to <2 mSv group (Table 1). Significant difference in the prevalence of high risk of psychological distress between the external radiation dose groups was observed in women, but not in men. Significant differences were found in the living place on March 11, 2011, housing status, evacuation, changes in work situation, and job loss^25^ between the three external radiation dose groups in both men and women (Table 1).

**Table 1.** Characteristics according to external radiation dose

		(missing)	<1 mSv	1 to <2 mSv	≥2 mSv	All	*P*
**All**							
Subjects, *n* (%)		27,783 (43.3)	25,412 (39.6)	8,424 (13.1)	2,565 (4.0)	64,184 (100)	
Men, *n* (%)		12,368 (44.5)	10,242 (40.3)	3,862 (45.8)	1,480 (57.7)	27,952 (43.5)	<0.001
Age, years, mean (SD)		55.0 (18.2)	53.7 (17.7)	54.5 (17.2)	53.9 (16.0)	54.4 (17.8)	<0.001
Living place on March 11, 2011, *n* (%)	: missing	273 (1.0)	52 (0.2)	2 (0.0)	0 (0.0)	327 (0.5)	<0.001
	: Hamadori	17,965 (64.7)	21,659 (85.2)	6,622 (78.6)	2,119 (82.6)	48,365 (75.4)	
	: Nakadori	9,536 (34.3)	3,694 (14.5)	1,800 (21.4)	446 (17.4)	15,476 (24.1)	
	: Aizu	9 (0.0)	7 (0.0)	0 (0.0)	0 (0.0)	16 (0.0)	
Housing status, *n* (%)	: own house	10,402 (46.8)	5,471 (28.2)	2,772 (41.6)	427 (20.2)	19,072 (37.8)	<0.001
	: evacuation shelter or temporary housing	2,192 (9.9)	3,058 (15.8)	1,002 (15.0)	308 (14.6)	6,560 (13.0)	
	: others	9,656 (43.4)	10,875 (56.0)	2,892 (43.4)	1,381 (65.3)	24,804 (49.2)	
Evacuation, *n* (%)		12,436 (44.8)	16,231 (63.9)	4,494 (53.3)	1,900 (74.1)	35,061 (54.6)	<0.001
Changes in work situation, *n* (%)		13,358 (52.2)	14,071 (58.9)	4,357 (54.9)	1,581 (64.4)	33,367 (55.7)	<0.001
Job loss, *n* (%)		4,923 (17.7)	6,244 (24.6)	1,735 (20.6)	527 (20.5)	13,429 (20.9)	<0.001
Psychological distress (K6 ≥13), *n* (%)		3,617 (14.2)	3,624 (15.1)	1,118 (14.0)	356 (15.0)	8,715 (14.6)	0.010
Probable PTSD: (PCL ≥44), *n* (%)		5,526 (21.4)	5,392 (22.1)	1,619 (20.1)	527 (21.7)	13,064 (21.5)	0.001
Perception of radiation risk to genetic effects, *n* (%): “likely” or “very likely”		15,500 (60.2)	14,418 (60.0)	4,867 (60.8)	1,432 (59.2)	36,217 (60.1)	0.43
Subjective health status, *n* (%)	: fine	5,044 (18.6)	4,297 (17.3)	1,443 (17.5)	413 (16.5)	11,197 (17.8)	<0.001
	: passably	17,220 (63.4)	15,883 (63.9)	5,276 (64.0)	1,592 (63.5)	39,971 (63.7)	
	: poor	4,890 (18.0)	4,693 (18.9)	1,530 (18.5)	502 (20.0)	11,615 (18.5)	

**Men**							
Subjects, *n* (%)		12,368 (44.2)	10,242 (36.6)	3,862 (13.8)	1,480 (5.3)	27,952 (100)	
Age, years, mean (SD)		55.8 (17.9)	55.4 (17.5)	55.9 (16.8)	52.9 (15.9)	55.5 (17.5)	<0.001
Living place on March 11, 2011, *n* (%)	: missing	105 (0.8)	23 (0.2)	1 (0.0)	0 (0.0)	129 (0.5)	<0.001
	: Hamadori	7,904 (63.9)	8,668 (84.6)	3,058 (79.2)	1,245 (84.1)	20,875 (74.7)	
	: Nakadori	4,354 (35.2)	1,550 (15.1)	803 (20.8)	235 (15.9)	6,942 (24.8)	
	: Aizu	5 (0.0)	1 (0.0)	0 (0.0)	0 (0.0)	6 (0.0)	
Housing status, *n* (%)	: own house	4,836 (48.1)	2,354 (30.0)	1,343 (44.0)	240 (19.5)	8,773 (39.5)	<0.001
	: evacuation shelter or temporary housing	1,024 (10.2)	1,324 (16.9)	456 (14.9)	188 (15.3)	2,992 (13.5)	
	: others	4,196 (41.7)	4,170 (53.1)	1,256 (41.1)	804 (65.3)	10,426 (47.0)	
Evacuation, *n* (%)		5,493 (44.4)	6,520 (63.7)	2,039 (52.8)	1,135 (76.7)	15,187 (54.3)	<0.001
Changes in work situation, *n* (%)		6,224 (53.3)	5,892 (60.0)	2,048 (55.1)	931 (64.6)	15,095 (56.6)	<0.001
Job loss, *n* (%)		1,927 (15.6)	2,225 (21.7)	730 (18.9)	260 (17.6)	5,142 (18.4)	<0.001
Psychological distress (K6 ≥13), *n* (%)		1,316 (11.4)	1,224 (12.6)	428 (11.6)	164 (11.7)	3,132 (11.9)	0.074
Probable PTSD: (PCL ≥44), *n* (%)		2,065 (17.6)	1,881 (19.0)	654 (17.5)	248 (17.4)	4,848 (18.1)	0.038
Perception of radiation risk to genetic effects, *n* (%): “likely” or “very likely”		6,573 (56.6)	5,447 (55.9)	2,125 (57.1)	748 (53.0)	14,893 (56.2)	0.042
Subjective health status, *n* (%)	: fine	2,719 (22.4)	2,028 (20.1)	773 (20.3)	309 (21.2)	5,829 (21.2)	<0.001
	: passably	7,387 (60.9)	6,201 (61.5)	2,349 (61.8)	905 (62.1)	16,842 (61.3)	
	: poor	2,030 (16.7)	1,848 (18.3)	677 (17.8)	243 (16.7)	4,798 (17.5)	

**Women**							
Subjects, *n* (%)		15,415 (42.5)	15,170 (41.9)	4,562 (12.6)	1,085 (3.0)	36,232 (100)	
Age, years, mean (SD)		54.3 (18.4)	52.5 (17.7)	53.3 (17.4)	55.2 (16.1)	53.5 (18.0)	<0.001
Living place on March 11, 2011, *n* (%)	: missing	168 (1.1)	29 (0.2)	1 (0.0)	0 (0.0)	198 (0.5)	<0.001
	: Hamadori	10,061 (65.3)	12,991 (85.6)	3,564 (78.1)	874 (80.6)	27,490 (75.9)	
	: Nakadori	5,182 (33.6)	2,144 (14.1)	997 (21.9)	211 (19.4)	8,534 (23.6)	
	: Aizu	4 (0.0)	6 (0.0)	0 (0.0)	0 (0.0)	10 (0.0)	
Housing status, *n* (%)	: own house	5,566 (45.6)	3,117 (27.0)	1,429 (39.6)	187 (21.2)	10,299 (36.5)	<0.001
	: evacuation shelter or temporary housing	1,168 (9.6)	1,734 (15.0)	546 (15.1)	120 (13.6)	3,568 (12.6)	
	: others	5,460 (44.8)	6,705 (58.0)	1,636 (45.3)	577 (65.3)	14,378 (50.9)	
Evacuation, *n* (%)		6,943 (45.0)	9,711 (64.0)	2,455 (53.8)	765 (70.5)	19,874 (54.9)	<0.001
Changes in work situation, *n* (%)		7,134 (51.2)	8,179 (58.2)	2,309 (54.7)	650 (64.0)	18,272 (55.0)	<0.001
Job loss, *n* (%)		2,996 (19.4)	4,019 (26.5)	1,005 (22.0)	267 (24.6)	8,287 (22.9)	<0.001
Psychological distress (K6 ≥13), *n* (%)		2,301 (16.4)	2,400 (16.9)	690 (16.2)	192 (19.7)	5,583 (16.7)	0.039
Probable PTSD: (PCL ≥44), *n* (%)		3,461 (24.4)	3,511 (24.3)	965 (22.3)	279 (27.8)	8,216 (24.2)	0.001
Perception of radiation risk to genetic effects, *n* (%): “likely” or “very likely”		8,927 (63.1)	8,971 (62.7)	2,742 (64.1)	684 (67.9)	21,324 (63.2)	0.006
Subjective health status, *n* (%)	: fine	2,325 (15.5)	2,269 (15.3)	670 (15.1)	104 (9.9)	5,368 (15.2)	<0.001
	: passably	9,833 (65.5)	9,682 (65.4)	2,927 (65.8)	687 (65.4)	23,129 (65.5)	
	: poor	2,860 (19.0)	2,845 (19.2)	853 (19.2)	259 (24.7)	6,817 (19.3)	

K6, Kessler’s 6-item Psychological Distress Scale; PCL, Post-Traumatic Stress Disorder Checklist; SD, standard deviation.

Table 2 shows the characteristics of the groups divided by the K6 or PCL cut-off scores (K6 score ≥13 and PCL score ≥44). Significant differences were found in the living place on March 11, 2011, housing status, evacuation, changes in work situation, loss of job, and risk perception for the next generation between the two groups divided by the K6 or PCL cut-off score (Table 2).

**Table 2.** Characteristics according to K6 score and PCL score

		All			Men			Women		
		
		K6 <13	K6 ≥13	*P*	K6 <13	K6 ≥13	*P*	K6 <13	K6 ≥13	*P*
Subjects, *n* (%)		51,088 (85.4)	8,715 (14.6)		23,188 (88.1)	3,132 (11.9)		27,900 (83.3)	5,583 (16.7)	
Age, years, mean (SD)		53.1 (17.7)	54.0 (17.2)	<0.001	54.7 (17.4)	53.8 (17.0)	0.01	51.8 (17.7)	54.0 (17.3)	<0.001
External radiation dose	: missing	21,891 (42.8)	3,617 (41.5)	0.010	10,180 (43.9)	1,316 (42.0)	0.07	11,711 (42.0)	2,301 (41.2)	0.039
	: <1 mSv	20,331 (39.8)	3,624 (41.6)		8,506 (36.7)	1,224 (39.1)		11,825 (42.4)	2,400 (43.0)	
	: 1 to <2 mSv	6,849 (13.4)	1,118 (12.8)		3,267 (14.1)	428 (13.7)		3,582 (12.8)	690 (12.4)	
	: ≥2 mSv	2,017 (3.9)	356 (4.1)		1,235 (5.3)	164 (5.2)		782 (2.8)	192 (3.4)	
Living place on March 11, 2011, *n* (%)	: missing	273 (0.5)	39 (0.4)	<0.001	111 (0.5)	11 (0.4)	<0.001	162 (0.6)	28 (0.5)	<0.001
	: Hamadori	37,786 (74.0)	7,404 (85.0)		17,064 (73.6)	2,633 (84.1)		20,722 (74.3)	4,771 (85.5)	
	: Nakadori	13,016 (25.5)	1,270 (14.6)		6,008 (25.9)	488 (15.6)		7,008 (25.1)	782 (14.0)	
	: Aizu	13 (0.0)	2 (0.0)		5 (0.0)	0 (0.0)		8 (0.0)	2 (0.0)	
Housing status, *n* (%)	: own house	16,194 (39.6)	1,679 (25.9)	<0.001	7,662 (41.1)	627 (26.4)	<0.001	8,532 (38.4)	1,052 (25.6)	<0.001
	: evacuation shelter or temporary housing	4,876 (11.9)	1,025 (15.8)		2,325 (12.5)	397 (16.7)		2,551 (11.5)	628 (15.3)	
	: others	19,799 (48.4)	3,780 (58.3)		8,648 (46.4)	1,355 (57.0)		11,151 (50.2)	2,425 (59.1)	
Evacuation, *n* (%)		27,015 (52.9)	5,769 (66.2)	<0.001	12,248 (52.8)	2,079 (66.4)	<0.001	14,767 (52.9)	3,690 (66.1)	<0.001
Changes in work situation, *n* (%)		25,785 (53.0)	5,676 (69.7)	<0.001	12,102 (54.1)	2,224 (73.6)	<0.001	13,683 (52.1)	3,452 (67.4)	<0.001
Job loss, *n* (%)		10,141 (19.9)	2,581 (29.6)	<0.001	3,940 (17.0)	906 (28.9)	<0.001	6,201 (22.2)	1,675 (30.0)	<0.001
Probable PTSD: (PCL ≥44), *n* (%)		5,904 (11.8)	6,362 (75.4)	<0.001	2,339 (10.3)	2,226 (72.8)	<0.001	3,565 (13.2)	4,136 (76.9)	<0.001
Perception of radiation risk to genetic effects, *n* (%): “likely” or “very likely”		27,308 (55.9)	6,775 (81.4)	<0.001	11,733 (52.6)	2,379 (78.8)	<0.001	15,575 (58.7)	4,396 (82.8)	<0.001
Subjective health status, *n* (%)	: fine	10,515 (20.9)	312 (3.7)	<0.001	5,484 (24.0)	145 (4.7)	<0.001	5,031 (18.4)	167 (3.1)	<0.001
	: passably	33,380 (66.5)	3,985 (46.8)		14,497 (63.4)	1,410 (45.8)		18,883 (69.0)	2,575 (47.4)	
	: poor	6,310 (12.6)	4,213 (49.5)		2,870 (12.6)	1,521 (49.4)		3,440 (12.6)	2,692 (49.5)	

		PCL <44	PCL ≥44	*P*	PCL <44	PCL ≥44	*P*	PCL <44	PCL ≥44	*P*

Subjects, *n* (%)		47,636 (78.5)	13,064 (21.5)		21,903 (81.9)	4,848 (18.1)		25,733 (75.8)	8,216 (24.2)	
Age, years, mean (SD)		52.5 (17.6)	56.5 (17.1)	<0.001	54.2 (17.4)	57.7 (16.8)	<0.001	51.1 (17.6)	55.9 (17.2)	<0.001
External radiation dose	: missing	20,346 (42.7)	5,526 (42.3)	0.001	9,638 (44.0)	2,065 (42.6)	0.038	10,708 (41.6)	3,461 (42.1)	0.001
	: <1 mSv	18,959 (39.8)	5,392 (41.3)		8,013 (36.6)	1,881 (38.8)		10,946 (42.5)	3,511 (42.7)	
	: 1 to <2 mSv	6,432 (13.5)	1,619 (12.4)		3,078 (14.1)	654 (13.5)		3,354 (13.0)	965 (11.7)	
	: ≥2 mSv	1,899 (4.0)	527 (4.0)		1,174 (5.4)	248 (5.1)		725 (2.8)	279 (3.4)	
Living place on March 11, 2011, *n* (%)	: missing	246 (0.5)	70 (0.5)	<0.001	106 (0.5)	19 (0.4)	<0.001	140 (0.5)	51 (0.6)	<0.001
	: Hamadori	34,938 (73.3)	10,983 (84.1)		15,988 (73.0)	4,049 (83.5)		18,950 (73.6)	6,934 (84.4)	
	: Nakadori	12,441 (26.1)	2,008 (15.4)		5,804 (26.5)	780 (16.1)		6,637 (25.8)	1,228 (14.9)	
	: Aizu	11 (0.0)	3 (0.0)		5 (0.0)	0 (0.0)		6 (0.0)	3 (0.0)	
Housing status, *n* (%)	: own house	15,536 (40.4)	2,628 (27.2)	<0.001	7,442 (41.9)	1,006 (27.7)	<0.001	8,094 (39.1)	1,622 (27.0)	<0.001
	: evacuation shelter or temporary housing	4,445 (11.6)	1,609 (16.7)		2,182 (12.3)	635 (17.5)		2,263 (10.9)	974 (16.2)	
	: others	18,469 (48.0)	5,409 (56.1)		8,119 (45.8)	1,989 (54.8)		10,350 (50.0)	3,420 (56.8)	
Evacuation, *n* (%)		24,780 (52.0)	8,545 (65.4)	<0.001	11,391 (52.0)	3,202 (66.0)	<0.001	13,389 (52.0)	5,343 (65.0)	<0.001
Changes in work situation, *n* (%)		23,613 (51.8)	8,374 (69.2)	<0.001	11,265 (53.1)	3,332 (72.4)	<0.001	12,348 (50.7)	5,042 (67.3)	<0.001
Job loss, *n* (%)		9,149 (19.2)	3,768 (28.8)	<0.001	3,579 (16.3)	1,359 (28.0)	<0.001	5,570 (21.6)	2,409 (29.3)	<0.001
Psychological distress (K6 ≥13), *n* (%)		2,074 (4.5)	6,362 (51.9)	<0.001	831 (3.9)	2,226 (48.8)	<0.001	1,243 (5.0)	4,136 (53.7)	<0.001
Perception of radiation risk to genetic effects, *n* (%): “likely” or “very likely”		24,555 (53.7)	10,167 (81.9)	<0.001	10,673 (50.6)	3,732 (80.3)	<0.001	13,882 (56.5)	6,435 (82.9)	<0.001
Subjective health status, *n* (%)	: fine	10,222 (21.8)	661 (5.2)	<0.001	5,370 (24.9)	310 (6.5)	<0.001	4,852 (19.2)	351 (4.4)	<0.001
	: passably	31,287 (66.7)	6,638 (52.3)		13,706 (63.5)	2,441 (51.4)		17,581 (69.5)	4,197 (52.8)	
	: poor	5,369 (11.5)	5,401 (42.5)		2,519 (11.7)	2,002 (42.1)		2,850 (11.3)	3,399 (42.8)	

K6, Kessler’s 6-item Psychological Distress Scale; PTSD, post-traumatic stress disorder; PCL, PTSD Checklist; SD, standard deviation.

### Association between external radiation doses and psychological distress

The ORs and 95% CIs for high risk of psychological distress (K6 score ≥13) according to external radiation doses are presented in Table 3. Compared with the <1 mSv group, the age- and sex-adjusted ORs for the 1 to <2 mSv and ≥2 mSv groups were 0.97 (95% CI, 0.91–1.04) and 1.08 (95% CI, 0.98–1.20), respectively. After controlling for all variables (age, sex, evacuation, perception of radiation risk, and subjective health status), the adjusted ORs for the 1 to <2 mSv and ≥2 mSv groups were 0.97 (95% CI, 0.90–1.04) and 0.98 (95% CI, 0.88–1.09), respectively. In women, the ≥2 mSv group tended to have a higher risk of psychological distress with the age-adjusted OR of 1.13 (95% CI, 0.99–1.30) compared to the <1 mSv group. After controlling for all the above listed variables., the adjusted OR for the ≥2 mSv group decreased to 1.00 (95% CI, 0.86–1.16).

**Table 3.** Odds ratios and 95% CIs of psychological distress (K6 ≥13) or post-traumatic stress (PCL ≥44) for external radiation dose among men and women

	K6 ≥13					PCL ≥44				
	
	<1 mSv	1 to <2 mSv		≥2 mSv		<1 mSv	1 to <2 mSv		≥2 mSv	
	
	Ref	OR	(95% CI)	OR	(95% CI)	Ref	OR	(95% CI)	OR	(95% CI)
**ALL**										
crude OR	Ref	0.94	(0.88–1.01)	1.01	(0.91–1.11)	Ref	0.91	(0.85–0.97)	0.98	(0.90–1.07)
model 1 OR	Ref	0.97	(0.91–1.04)	1.08	(0.98–1.20)	Ref	0.94	(0.88–0.997)	1.07	(0.98–1.17)
model 2 OR	Ref	1.00	(0.93–1.07)	1.00	(0.91–1.11)	Ref	0.96	(0.90–1.03)	0.98	(0.90–1.07)
model 3 OR	Ref	0.96	(0.90–1.03)	1.08	(0.97–1.19)	Ref	0.92	(0.86–0.99)	1.05	(0.96–1.16)
model 4 OR	Ref	0.96	(0.89–1.03)	1.02	(0.92–1.14)	Ref	0.92	(0.86–0.99)	1.01	(0.92–1.11)
model 5 OR	Ref	0.99	(0.92–1.06)	1.00	(0.91–1.11)	Ref	0.95	(0.88–1.02)	0.98	(0.89–1.07)
model 6 OR	Ref	0.97	(0.90–1.04)	0.98	(0.88–1.09)	Ref	0.93	(0.86–0.997)	0.95	(0.86–1.05)

**Men**										
crude OR	Ref	0.96	(0.87–1.07)	1.02	(0.89–1.18)	Ref	0.94	(0.87–1.02)	0.98	(0.84–1.14)
model 1 OR	Ref	0.96	(0.87–1.07)	1.01	(0.88–1.17)	Ref	0.95	(0.87–1.03)	1.01	(0.87–1.18)
model 2 OR	Ref	0.99	(0.89–1.10)	0.94	(0.81–1.08)	Ref	0.98	(0.90–1.06)	0.93	(0.80–1.08)
model 3 OR	Ref	0.95	(0.85–1.06)	1.03	(0.89–1.18)	Ref	0.93	(0.86–1.02)	1.02	(0.88–1.19)
model 4 OR	Ref	0.95	(0.85–1.06)	0.98	(0.84–1.14)	Ref	0.94	(0.86–1.02)	0.99	(0.84–1.15)
model 5 OR	Ref	0.98	(0.88–1.09)	0.95	(0.83–1.10)	Ref	0.97	(0.89–1.05)	0.94	(0.80–1.09)
model 6 OR	Ref	0.95	(0.85–1.07)	0.95	(0.81–1.10)	Ref	0.95	(0.87–1.04)	0.93	(0.80–1.09)

**Women**										
crude OR	Ref	0.98	(0.90–1.06)	1.13	(0.99–1.30)	Ref	0.93	(0.85–1.01)	1.12	(0.99–1.27)
model 1 OR	Ref	0.98	(0.90–1.06)	1.13	(0.99–1.30)	Ref	0.93	(0.85–1.01)	1.12	(0.99–1.27)
model 2 OR	Ref	1.00	(0.92–1.09)	1.06	(0.93–1.21)	Ref	0.95	(0.86–1.04)	1.04	(0.91–1.18)
model 3 OR	Ref	0.97	(0.89–1.05)	1.11	(0.97–1.27)	Ref	0.91	(0.83–1.01)	1.09	(0.96–1.24)
model 4 OR	Ref	0.97	(0.89–1.06)	1.04	(0.90–1.21)	Ref	0.91	(0.82–1.003)	1.03	(0.90–1.18)
model 5 OR	Ref	0.99	(0.91–1.08)	1.04	(0.91–1.19)	Ref	0.93	(0.85–1.03)	1.01	(0.89–1.15)
model 6 OR	Ref	0.98	(0.89–1.07)	1.00	(0.86–1.16)	Ref	0.91	(0.82–1.01)	0.97	(0.84–1.11)

CI, confidence interval; K6, Kessler’s 6-item Psychological Distress Scale; OR, odds ratio; PCL, Post-Traumatic Stress Disorder Checklist.

model 1: age, sex adjusted.

model 2: model 1 + evacuation adjusted.

model 3: model 1 + perception of radiation risk adjusted.

model 4: model 1 + subjective health status adjusted.

model 5: model 2 + perception of radiation risk adjusted.

model 6: model 5 + subjective health status adjusted.

Table 4 shows the ORs and 95% CIs for high risk of psychological distress according to external radiation doses without multiple imputation. When imputation was not performed, the age- and sex-adjusted ORs for 1 to <2 mSv and ≥2 mSv groups were 0.93 (95% CI, 0.87–1.001) and 1.06 (95% CI, 0.94–1.20), respectively. After adjusting for all variables, the adjusted ORs for the 1 to <2 mSv and ≥2 mSv groups were 0.95 (95% CI, 0.88–1.03) and 0.99 (95% CI, 0.87–1.13), respectively. In women, although the age-adjusted OR for the ≥2 mSv group compared to the <1 mSv group was 1.19 (95% CI, 1.01–1.40), the adjusted OR for the ≥2 mSv group was 1.05 (95% CI, 0.87–1.25) after the adjusting for all the above listed variables.

**Table 4.** Odds ratios and 95% CIs of psychological distress (K6 ≥13) or post-traumatic stress (PCL ≥44) for external radiation dose among men and women (without multiple imputation)

	K6 ≥13					PCL ≥44				
	
	<1 mGy	1 to <2 mGy		≥2 mGy		<1 mGy	1 to <2 mGy		≥2 mGy	
	
	Ref	OR	(95% CI)	OR	(95% CI)	Ref	OR	(95% CI)	OR	(95% CI)
**ALL**										
crude OR	Ref	0.92	(0.85–0.99)	0.99	(0.88–1.11)	Ref	0.89	(0.83–0.94)	0.98	(0.88–1.08)
model 1 OR	Ref	0.93	(0.87–1.001)	1.06	(0.94–1.20)	Ref	0.89	(0.84–0.95)	1.04	(0.94–1.16)
model 2 OR	Ref	0.98	(0.91–1.06)	1.02	(0.90–1.14)	Ref	0.94	(0.89–1.01)	0.99	(0.90–1.10)
model 3 OR	Ref	0.92	(0.85–0.99)	1.05	(0.93–1.19)	Ref	0.88	(0.82–0.93)	1.03	(0.93–1.14)
model 4 OR	Ref	0.93	(0.86–1.01)	1.02	(0.89–1.15)	Ref	0.89	(0.83–0.95)	1.00	(0.89–1.11)
model 5 OR	Ref	0.97	(0.90–1.04)	1.01	(0.90–1.14)	Ref	0.93	(0.87–0.99)	0.99	(0.89–1.10)
model 6 OR	Ref	0.95	(0.88–1.03)	0.99	(0.87–1.13)	Ref	0.91	(0.85–0.97)	0.96	(0.86–1.08)

**Men**										
crude OR	Ref	0.91	(0.81–1.02)	0.92	(0.78–1.10)	Ref	0.91	(0.82–0.999)	0.90	(0.78–1.04)
model 1 OR	Ref	0.91	(0.81–1.03)	0.92	(0.77–1.09)	Ref	0.90	(0.81–0.99)	0.94	(0.81–1.09)
model 2 OR	Ref	0.96	(0.85–1.08)	0.87	(0.73–1.03)	Ref	0.96	(0.87–1.06)	0.88	(0.76–1.02)
model 3 OR	Ref	0.90	(0.80–1.01)	0.95	(0.79–1.13)	Ref	0.88	(0.80–0.98)	0.96	(0.82–1.11)
model 4 OR	Ref	0.91	(0.80–1.03)	0.93	(0.77–1.11)	Ref	0.90	(0.81–1.001)	0.94	(0.81–1.10)
model 5 OR	Ref	0.94	(0.84–1.06)	0.90	(0.75–1.07)	Ref	0.94	(0.85–1.04)	0.90	(0.77–1.05)
model 6 OR	Ref	0.91	(0.80–1.04)	0.91	(0.76–1.10)	Ref	0.92	(0.83–1.03)	0.92	(0.78–1.07)

**Women**										
crude OR	Ref	0.95	(0.87–1.04)	1.21	(1.03–1.43)	Ref	0.90	(0.83–0.97)	1.20	(1.04–1.39)
model 1 OR	Ref	0.94	(0.86–1.03)	1.19	(1.01–1.40)	Ref	0.88	(0.81–0.96)	1.16	(1.000–1.33)
model 2 OR	Ref	1.00	(0.91–1.09)	1.15	(0.98–1.36)	Ref	0.93	(0.86–1.01)	1.12	(0.97–1.30)
model 3 OR	Ref	0.93	(0.85–1.02)	1.14	(0.96–1.35)	Ref	0.87	(0.80–0.95)	1.11	(0.95–1.28)
model 4 OR	Ref	0.95	(0.86–1.05)	1.08	(0.90–1.28)	Ref	0.88	(0.80–0.96)	1.05	(0.90–1.22)
model 5 OR	Ref	0.98	(0.89–1.08)	1.11	(0.94–1.31)	Ref	0.91	(0.84–0.99)	1.07	(0.93–1.25)
model 6 OR	Ref	0.97	(0.88–1.08)	1.05	(0.87–1.25)	Ref	0.89	(0.82–0.97)	1.01	(0.86–1.18)

CI, confidence interval; K6, Kessler’s 6-item Psychological Distress Scale; OR, odds ratio; PCL, Post-Traumatic Stress Disorder Checklist.

model 1: age, sex adjusted.

model 2: model 1 + evacuation adjusted.

model 3: model 1 + perception of radiation risk adjusted.

model 4: model 1 + subjective health status adjusted.

model 5: model 2 + perception of radiation risk adjusted.

model 6: model 5 + subjective health status adjusted.

eTable 1 shows the ORs and 95% CIs of psychological distress according to two models adjusted for evacuation. In the model 6 (age, sex, evacuation, perception of radiation risk, and subjective health status adjusted), the ORs for evacuation (reference: no evacuation), perception of radiation risk: high (reference: low), and subjective health status: poor (reference: fine) were 1.40 (95% CI, 1.33–1.48), 2.67 (95% CI, 2.51–2.83), and 18.54 (95% CI, 16.41–20.95), respectively (eTable 1).

### Association between external radiation doses and post-traumatic stress

For post-traumatic stress, the age- and sex-adjusted ORs for the 1 to <2 mSv and ≥2 mSv groups compared with the <1 mSv group were 0.94 (95% CI, 0.88–0.997) and 1.07 (95% CI, 0.98–1.17), respectively (Table 3). After controlling for all variables (age, sex, evacuation, perception of radiation risk, and subjective health status), the adjusted ORs for the 1 to <2 mSv and ≥2 mSv groups were 0.93 (95% CI, 0.86–0.997) and 0.95 (95% CI, 0.86–1.05), respectively (Table 3).

When imputation was not performed, the age- and sex-adjusted ORs for the 1 to <2 mSv and ≥2 mSv groups were 0.89 (95% CI, 0.84–0.95) and 1.04 (95% CI, 0.94–1.16), respectively. After adjusting for all the above listed variables, the adjusted ORs for the 1 to <2 mSv and ≥2 mSv groups were 0.91 (95% CI, 0.85–0.97) and 0.96 (95% CI, 0.86–1.08), respectively (Table 4). In women, the age-adjusted OR for the 1 to <2 mSv and ≥2 mSv groups compared to the <1 mSv group were 0.88 (95% CI, 0.81–0.96) and 1.16 (95% CI, 1.00–1.33), respectively. The adjusted OR for the 1 to <2 mSv and ≥2 mSv groups compared to the <1 mSv group were 0.89 (95% CI, 0.82–0.97) and 1.01 (95% CI, 0.86–1.18) after controlling for all variables, respectively (Table 4).

eTable 1 shows the ORs and 95% CIs of post-traumatic stress according to two models adjusted for evacuation. In the model 6 (age, sex, evacuation, perception of radiation risk, and subjective health status adjusted), the ORs for evacuation (reference: no evacuation), perception of radiation risk: high (reference: low), and subjective health status: poor (reference: fine) were 1.52 (95% CI, 1.45–1.59), 3.23 (95% CI, 3.06–3.40), and 10.63 (95% CI, 9.72–11.64), respectively (eTable 1).

## DISCUSSION

To our knowledge, this is the first study to investigate the association between individual external radiation doses and psychological distress or post-traumatic stress with a large sample size after the Fukushima Daiichi nuclear power plant accident. In this study with a total of 64,184 subjects, we found that women in the ≥2 mSv group tended to have a higher risk of psychological distress; however, the association became non-significant after adjusting for all confounding variables. On the other hand, there were no dose-dependent associations between radiation dose and post-traumatic stress.

In this study, although the women in the ≥2 mSv group tended to have a higher risk of psychological distress with the age-adjusted OR of 1.13 (95% CI, 0.99–1.30) before controlling variables, the adjusted OR decreased to 1.00 (95% CI, 0.86–1.16) after adjustment for evacuation, perception of radiation risk to genetic effects, and subjective health status. Evacuation, perception of radiation risk, and subjective health status were associated with psychological distress in this study (eTable 1). It is supposed that women in the ≥2 mSv group were exposed to stressful situations, such as evacuation and movement, and might be anxious about the risk of radiation. Many studies have suggested that women have a greater tendency to express emotions such as fear after traumatic events,^11^^,^^26^^,^^27^ and are less likely to use positive coping strategies.^28^ Taken together, our results of the association between external radiation doses and psychological distress in women suggest that stressful situations and/or anxiety about the risk of radiation may increase psychological distress in women in the ≥2 mSv group. Although a few studies have investigated the relationship between environmental radiation doses and mental health status after the Chernobyl nuclear disaster,^29^^,^^30^ the results are inconsistent. A previous study^9^ indicated that psychological distress in each evacuation zone was significantly positively associated with radiation levels in the environment after the Fukushima Daiichi nuclear power plant accident. However, that study used environmental radiation levels in the evacuation zone and may not accurately reflect people’s movement or evacuation from their original locations after the accident. In the present study, individual external radiation doses were estimated based on their behavioral data and daily gamma ray dose rate maps drawn after the accident, which could reflect the actual condition. Our results suggest no association between individual external radiation doses and psychological distress and is consistent with another cross-sectional study,^31^ which indicated that environmental radiation levels were not significantly associated with psychological distress 5 years after the earthquake. Along with various risk factors, including personal, socioeconomic, and disaster-related factors, perception of radiation risk was associated with psychological distress after the Fukushima Daiichi nuclear power plant accident.^10^ Together with the null findings in this study, evacuation, risk perception^10^ and anxiety regarding radiation^31^ might be more important factors for psychological distress than external radiation doses.

Although the 1 to <2 mSv group was associated with a lower risk of post-traumatic stress after controlling for variables, there were no dose-dependent associations between radiation dose and post-traumatic stress. Other factors, such as disaster-related or socioeconomic factors, might have affected the results of the association between the 1 to <2 mSv group and lower risk. A previous study^32^ showed that chronic diseases (physical or mental), worries about livelihood, lost jobs, lost social ties, and concerns about compensation were significant predictors of probable PTSD 1 year after the Fukushima Daiichi nuclear power plant accident. In the present study, the proportion of subjects who experienced evacuation was significantly different among the three radiation dose groups, with the lowest in the 1 to <2 mSv group (53.3%, <1 mSv group: 63.9%, ≥2 mSv groups: 74.1%). Furthermore, evacuation was significantly associated with post-traumatic stress (PCL score ≥44) (eTable 1). The difference in experience of evacuation, including housing arrangements, might have a considerable effect on post-traumatic stress.

This study has some potential limitations. First, although we imputed external radiation doses using multiple imputation,^22^ the response rate to the self-administered questionnaire regarding external doses was relatively low (56.7%). There might be a possibility that some response bias could have an influence on this rate. However, the results without multiple imputation showed similar associations, which supports our findings. Second, external doses were estimated using a self-administered questionnaire, and it was possible that the inaccuracy of the answers affects classification in the external radiation groups. Third, there might be residents who were in a bad mental health status and could not answer the survey. Previous studies^33^^,^^34^ indicated that mental health status might affect the response rate to the survey, which might have occurred in the present study. Fourth, we did not examine the relationship between internal radiation doses and psychological distress or post-traumatic stress. Fifth, high level of psychological distress might affect subjective health status in the study participants. More frequent poor subjective health status could be a consequence of high level of psychological distress, which may lead to over-adjustment of ORs in the results of this study. Finally, insufficient information was available regarding pre-disaster factors, such as personality traits, social adaptation, traumatic experiences, and other mental health problems.^11^ Nevertheless, to our knowledge, this is the first large-scale and systematic survey among evacuees to investigate the association between external radiation doses and psychological distress or post-traumatic stress after a nuclear accident. Our findings of no associations between external radiation doses and psychological distress provide important evidence for evacuees after nuclear accidents.

In conclusion, although the women in the ≥2 mSv group tended to have higher risk of psychological distress, the adjusted OR decreased to 1.00 (95% CI, 0.86–1.16) after controlling for variables. Although the 1 to <2 mSv group was associated with a lower risk of post-traumatic stress after controlling for variables, there were no dose-dependent associations between radiation dose and post-traumatic stress. Evacuation and risk perception of radiation may increase psychological distress in women in the higher radiation dose group. Since evacuees have different backgrounds and experiences after a nuclear disaster, careful assessment of various factors, including disaster-related factors and risk perception, is needed for individual interventions. Moreover, since mental health problems, such as psychological distress, post-traumatic stress, and radiation health anxiety, exist for a long time, further mid- and long-term studies are needed to confirm and extend the results.

## SUPPLEMENTARY MATERIAL

eTable 1. Odds ratios and 95% CIs of psychological distress (K6 ≥13) or post-traumatic stress (PCL ≥44) according to two models adjusted for evacuation status among men and women
