# Supplementary material for: Associations Between External Radiation Doses and the Risk of Psychological Distress or Post-traumatic Stress After the Fukushima Daiichi Nuclear Power Plant Accident: the Fukushima Health Management Survey
Source: J Epidemiol. 2022 Dec 5;32(Suppl 12):S95–S103. doi: 10.2188/jea.JE20210226 (PMC9703924; doi:10.2188/jea.JE20210226)
Supplement: Supplementary file 1 [file je-32-S095-s001.pdf]

eTable 1. Odds ratios and 95% CIs of psychological distress (K6 ≥13) or post-traumatic stress (PCL ≥44) according to two models adjusted for evacuation status among men and women

	K6 ≥13				PCL ≥44			
	Model 2		Model 6		Model 2		Model 6	
	OR	(95% CI)	OR	(95% CI)	OR	(95% CI)	OR	(95% CI)
**ALL**								
Radiation dose: 1 to <2 mSv (ref: <1 mSv)	1.00	(0.93–1.07)	0.97	(0.90–1.04)	0.96	(0.90–1.03)	0.93	(0.86–1.00)
Radiation dose: ≥2 mSv (ref: <1 mSv)	1.00	(0.91–1.11)	0.98	(0.88–1.09)	0.98	(0.90–1.07)	0.95	(0.86–1.05)
Sex: women (ref: men)	1.50	(1.43–1.57)	1.35	(1.28–1.42)	1.50	(1.44–1.56)	1.35	(1.29–1.41)
Age (years: continuous)	1.00	(1.00–1.01)	0.99	(0.99–0.99)	1.02	(1.01–1.02)	1.01	(1.01–1.01)
Evacuation (ref: no evacuation)	1.76	(1.68–1.85)	1.40	(1.33–1.48)	1.81	(1.74–1.89)	1.52	(1.45–1.59)
Perception of radiation risk: high (ref: low)			2.67	(2.51–2.83)			3.23	(3.06–3.40)
Subjective health status: passably (ref: fine)			3.58	(3.18–4.03)			2.58	(2.37–2.81)
Subjective health status: poor (ref: fine)			18.54	(16.41–20.95)			10.63	(9.72–11.64)
**Men**								
Radiation dose: 1 to <2 mSv (ref: <1 mSv)	0.99	(0.89–1.10)	0.95	(0.85–1.07)	0.98	(0.90–1.06)	0.95	(0.87–1.04)
Radiation dose: ≥2 mSv (ref: <1 mSv)	0.94	(0.81–1.08)	0.95	(0.81–1.10)	0.93	(0.80–1.08)	0.93	(0.80–1.09)
Age (years: continuous)	1.00	(1.00–1.00)	0.99	(0.99–0.99)	1.01	(1.01–1.02)	1.01	(1.00–1.01)
Evacuation (ref: no evacuation)	1.76	(1.63–1.91)	1.43	(1.31–1.55)	1.87	(1.75–2.00)	1.60	(1.49–1.72)
Perception of radiation risk: high (ref: low)			2.62	(2.38–2.88)			3.36	(3.10–3.64)
Subjective health status: passably (ref: fine)			3.45	(2.90–4.12)			2.50	(2.21–2.84)
Subjective health status: poor (ref: fine)			17.81	(14.86–21.35)			9.70	(8.50–11.08)
**Women**								
Radiation dose: 1 to <2 mSv (ref: <1 mSv)	1.00	(0.92–1.09)	0.98	(0.89–1.07)	0.95	(0.86–1.04)	0.91	(0.82–1.01)
Radiation dose: ≥2 mSv (ref: <1 mSv)	1.06	(0.93–1.21)	1.00	(0.86–1.16)	1.04	(0.91–1.18)	0.97	(0.84–1.11)
Age (years: continuous)	1.01	(1.01–1.01)	1.00	(1.00–1.00)	1.02	(1.02–1.02)	1.01	(1.01–1.01)
Evacuation (ref: no evacuation)	1.76	(1.65–1.87)	1.39	(1.30–1.48)	1.78	(1.69–1.87)	1.47	(1.39–1.55)
Perception of radiation risk: high (ref: low)			2.70	(2.50–2.92)			3.14	(2.93–3.35)

Subjective health status: passably (ref: fine)	3.70	(3.15–4.34)	2.65	(2.36–2.98)
Subjective health status: poor (ref: fine)	19.22	(16.28–22.69)	11.42	(10.10–12.91)

CI, confidence interval; K6, Kessler's 6-item Psychology Distress Scale; OR, odds ratio; PCL, Post-Traumatic Stress Disorder Checklist.

model 2: age, sex, and evacuation adjusted

model 6: age, sex, evacuation, perception of radiation risk, and subjective health status adjusted
